# Clinical evaluation of outdoor cats exposed to ectoparasites and associated risk for vector-borne infections in southern Italy

**DOI:** 10.1186/s13071-018-2725-8

**Published:** 2018-03-20

**Authors:** Maria Flaminia Persichetti, Maria Grazia Pennisi, Angela Vullo, Marisa Masucci, Antonella Migliazzo, Laia Solano-Gallego

**Affiliations:** 1 Istituto Zooprofilattico Sperimentale della Sicilia, A. Mirri, Via G. Marinuzzi 3, 90129 Palermo, Italy; 2 Dipartimento di Scienze Veterinarie, Università degli Studi di Messina, Polo Universitario Annunziata, 98168 Messina, Italy; 3 Departament de Medicina i Cirurgia Animals, Facultat de Veterinària, Universitat Autònoma de Barcelona, Bellaterra, Cerdanyola, 08193 Barcelona, Spain

**Keywords:** Cat, Vector-borne pathogens, Zoonosis, Risk factor, Ectoparasite, Outdoor lifestyle, Indoor lifestyle

## Abstract

**Background:**

Cats can be carriers of infected arthropods and be infected with several vector-borne pathogens (VBP) but there is limited knowledge about their pathogenic role in cats.

**Results:**

A cross-sectional controlled study investigated the clinical status and antibody (*Bartonella henselae*, *Rickettsia conorii*, *Ehrlichia canis*, *Anaplasma phagocytophilum*, *Babesia microti* and *Leishmania infantum*) and/or blood PCR (*Mycoplasma* spp., *Bartonella* spp., *Rickettsia* spp., *Ehrlichia/Anaplasma* spp., piroplasmids, *L. infantum*, *Hepatozoon felis*) prevalence in 197 cats. Outdoor cats lacking ectoparasiticide treatment or hosting ectoparasites (study group [SG], *n* = 134) and indoor cats treated against ectoparasites (control group [CG], *n* = 63) were enrolled. Clinical data and retroviral co-infections were compared between the two groups. Multivariable analysis tested associations between variables and VBP exposure. Lymphadenia, stomatitis, and various haematological abnormalities were statistically more frequent in SG. Antibodies against *R. conorii*, *B. henselae*, *A. phagocytophylum*, *B. microti*, *E. canis* and *L. infantum* were detected. *Bartonella henselae*, *Bartonella clarridgeiae*, *Mycoplasma haemofelis*, “*Candidatus* Mycoplasma haemominutum” and “*Candidatus* Mycoplasma turicensis” DNA were identified. Very high antibody (87.8%) and PCR (40.1%) positivity to at least one pathogen were detected and were significantly higher in SG. Co-infections were confirmed in about one-third of the cats and were more frequent in SG cats. Molecular and overall (antibody and PCR) positivity to *Bartonella* and antibody positivity to *R. conorii* were higher in SG. Multivariable analysis found significant associations of *Bartonella* spp. infection with Feline Immunodeficiency Virus (FIV) infection and increased globulins, and of *Mycoplasma* spp. infection with adult age, FIV infection, anaemia, and increased creatinine.

**Conclusions:**

A very high prevalence of exposure to zoonotic VBP was found in cats, with *Rickettsia* and *Bartonella* infections being most prevalent. Some risk factors were documented namely for *Mycoplasma* spp. and *Bartonella* spp. The lifestyle of cats is clinically relevant and requires specific preventative measures to protect their health.

**Electronic supplementary material:**

The online version of this article (10.1186/s13071-018-2725-8) contains supplementary material, which is available to authorized users.

## Background

Vector-borne infections (VBI) are caused by parasites, bacteria or viruses transmitted by hematophagous arthropods, and many of them are of zoonotic concern [1–6]. Cats have a high likelihood of ectoparasite exposures when living an outdoor lifestyle and there is a lack of preventive treatment with acaricides. Consequently, these animals can be carriers of infected arthropods and be infected with several vector-borne pathogens (VBP), as observed in dogs [1, 3–5, 7–11]. The lack of knowledge on the pathogenic role of most of these VBP in cats may limit the diagnosis of vector-borne diseases (VBD). Also, clinical signs and laboratory abnormalities associated with VBD are widely variable and non-specific [1, 3, 4]. Moreover, concurrent VBI or retroviral infections can be found, which may influence the clinical course and outcome of VBD in cats [3, 12].

Recent literature highlighted some risk factors associated with cat positivity to VBI such as multi-cat household, outdoor access, male gender, FIV positivity, and abortive FeLV infection [3, 4, 6, 12]. Preventative control measures against ectoparasites infestation, i.e. regular individual use of ectoparasiticide formulations, seem the most effective tool to prevent infection in cats, and other hosts [3, 4, 12]. The present controlled study evaluated the prevalence and risk factors for some VBP in cats exposed to ectoparasites in southern Italy and assessed the impact of the infections on their health status.

## Methods

### Study sites, cat enrolment and sampling procedures

A total of 197 cats were enrolled from March 2012 to March 2013 at four veterinary clinics located in Sicily (*n* = 39) (Veterinary Teaching Hospital, Università degli Studi di Messina, Messina and Ambulatorio Veterinario S. Lucia, Lipari-Messina) and Calabria (*n* = 158) (Clinica Veterinaria Camagna, Reggio Calabria and Ambulatorio Dr Cardone, Gioia Tauro-Reggio Calabria). Cats aged > 6 months and experiencing at least an intere vector-season since birth (April-October) were recruited irrespective of breed and gender. Most cats (*n* = 144; 73%) were admitted for elective surgery or annual health check. They were enrolled when the following information was available: type of housing and lifestyle and individual application of ectoparasiticides. According to this information and the occurrence of ectoparasites at physical examination, two groups of cats were considered. The study group (SG, *n* = 134) included cats with a greater chance of exposure to ectoparasites, i.e. outdoor cats with a lack of regular individual ectoparasiticide treatment and having ectoparasites at enrolment. The control group (CG, *n* = 63) was composed of indoor cats with no evidence of ectoparasites at enrolment, receiving the appropriate ectoparasiticide treatment, and therefore with a low risk for ectoparasites. Cats living in rescue catteries were excluded from this study.

Clinical history and physical examination findings for cats were registered in a clinical form. Also, information on region, age, sex, breed, lifestyle, ectoparasiticide treatments, flea and tick presence was included. Cats were classified as “young” if they had experienced only one vector-season since birth, and “adult” if they had experienced more than one vector-season.

From each cat, blood, conjunctival and oral swabs were obtained. One millilitre of blood was placed into one tube with EDTA and used within 24 hours for complete blood count (CBC) and subsequently stored at -20 °C until further use for molecular investigations. Left-over blood (about 2 ml) was used to perform blood smears (immediately) and to obtain serum after clotting into a dry tube. Blood serum was stored at -20 °C until further use for haematological and serological investigations. Urine samples were obtained by cystocentesis when possible and used for urinalysis within 2 hours and urine protein and creatinine ratio (UPC) within 24 h from collection. When enlarged lymph nodes were observed, a fine needle aspiration was performed. Sealed needles and swabs were stored at -20 °C until further use for molecular tests.

### Hematological investigations and urinalysis

Complete blood count was performed using a laser haematology analyser (IDEXX ProCyte Dx® Hematology Analyser, Idexx Laboratories, Westbrook, Maine, USA). Reference intervals of CBC are listed in Additional file 1: Table S1. Blood smears were stained by May Grünwald-Giemsa staining and examined for haematological abnormalities and presence of hemoparasites [13].

A biochemical profile including creatine kinase (CK), aspartate aminotransferase (AST), alanine aminotransferase (ALT), alkaline phosphatase (ALP), gamma glutamyl transferase (GGT), cholinesterase, total bilirubin, total proteins, albumin, globulin, albumin/globulin ratio, cholesterol, triglycerides, urea, creatinine, glucose, calcium, phosphorus, magnesium, sodium, potassium, sodium/potassium ratio, chloride, correct chloride, serum iron, unsatured iron-binding capacity (UIBC), total iron-binding capacity (TIBC), transferrin saturation, and serum amyloid A (SAA) was performed at the Laboratorio di analisi veterinarie San Marco (Padova, Italy) and reference intervals of the above parameters are shown in Additional file 2: Table S2.

Urinalysis was performed using Combur 9 Test strips (Roche Diagnostics, Indianapolis, Indiana, USA), Vet 360 refractometer (Reichert, Seefeld, Germany) and a microscopic evaluation of urine sediment was performed using the Kova system (Kova International, Garden Grove, CA, USA). Urine protein and creatinine ratio were evaluated with Catalyst Dx® Chemistry Analyzer (Idexx Laboratories, Westbrook, Maine, USA), using 0.4 as the cut-off value for proteinuria [14].

### Serological investigations

All cats were tested for feline leukaemia virus (FeLV) antigen and antibodies to feline immunodeficiency virus (FIV) by a rapid enzyme-linked immunosorbent assay (ELISA) (SNAP Combo Plus FeLV ag/FIV ab test, Idexx Laboratories, Westbrook, Maine, USA). Sera from individual cats were also tested for the presence of immunoglobulin G (IgG) antibodies against *Bartonella henselae*, *Rickettsia conorii*, *Ehrlichia canis*, *Babesia microti* and *Anaplasma phagocytophilum* antigens by the immunofluorescence antibody test (IFAT) using commercial kits (Fuller Laboratories Fullerton, California, USA). The manufacturer’s protocol was followed for all serological tests using a cut-off dilution of 1:64 for *B. henselae*, *R. conorii* and *B. microti*; and 1:50 for *E. canis* and *A. phagocytophilum*. The presence of *L. infantum* IgG antibodies was investigated by IFAT according to Persichetti et al. [15] and the cut-off value was established at 1:80 as previously described [15]. Titres of positive samples were determined for all pathogens, with the exception of *B. microti*, and considered high when they were at least four times over cut-off value.

### Molecular investigations

Quantitative real-time PCR (qPCR) technology and sequencing were applied as described elsewhere [10], to detect specific DNA markers for *Ehrlichia/Anaplasma* spp., piroplasmids (*Babesia* spp. and *Theileria* spp.), *Hepatozoon felis*, hemotropic *Mycoplasma* spp., *Rickettsia* spp., *Bartonella* spp. and *L. infantum* from feline blood samples, while infections by the latter pathogen were also diagnosed from conjunctival and oral swabs, urine, and lymph node specimens. Species-specific qPCRs were also performed as described by Martinez et al. [16] to discriminate among feline hemoplasmas species (*Mycoplasma haemofelis*, “*Candidatus* Mycoplasma haemominutum” and “*Candidatus* Mycoplasma turicensis”).

### Statistical analysis

The Kolmogorov-Smirnov normality test was used to assess the normality of distribution of the continuous variable age. Statistical differences between SG and CG cats were tested for significance by Fisher’s exact test for categorical variables and with the Mann-Whitney test for numerical variables using the GraphPad InStat v3.05 for Windows 95 (GraphPad Software Inc., San Diego California, USA, 2000). Differences were considered significant if the *P*-value was < 0.05. Independent variables that yielded significant differences between the 2 groups were analysed in the overall 197 cats with multivariable logistic regression analysis using the STATA 9.2 software (StataCorp LP, College Station, Texas) to test for possible risk factors associated with investigated VBP. According to the molecular and antibody detection, outcome variables were as follows: *L. infantum* (molecular and antibody tests), *Bartonella* spp. (molecular and antibody tests), *Mycoplasma* spp. (molecular test), *R. conorii* (antibody test), *E. canis* (antibody test), *B. microti* (antibody test), *A. phagocytophilum* (antibody test). Dichotomous variables (outcome variable and investigated risk factors) were analysed with multivariable logistic regression analysis, which was applied for each pathogen. Also, an ordered logistic regression model was applied to the variable response co-infection (i.e. exposure to more than one VBP) and the same potential risk factors were considered for single pathogens. The variable co-infection was measured on an ordinal scale as it considers the number of infections present simultaneously in a cat under investigation (no infection, one infection, ≥ 2 infections). *P*-values with an odds ratio (OR) and 95% confidence interval (CI) of multivariable analyses were obtained. An OR value > 1 implies a positive association between independent and dependent variables, while an OR < 1 implies an inverse association.

## Results

### Clinical evaluation

Cats were aged between 5 months and 19 years (median 2 years, 25th percentile 1 year, 75th percentile 5 years); 69 (35%) were males and 128 females (65%); eight pedigree cats were enrolled (five Persians and three Carthusians in the CG). The SG median age (2 years, 25th percentile 0.9 years, 75th percentile 4 years) was significantly lower compared to that of the CG (3 years, 25th percentile 1 year, 75th percentile 8 years) (Mann-Whitney *U-*test: *U*_(195)_= 3387, *Z* = -2.23327, *P* = 0.0257). Moreover, a significantly higher percentage of SG cats (79%) were admitted for elective surgery or annual health check compared to the CG (60%) (Fisher’s exact test: *P* = 0.0093, OR = 2.49, 95% CI:1.29–7.79). One or more abnormalities were observed in all cats at a physical examination or laboratory investigations (CBC, biochemical profile and urinalysis); therefore, no cat was considered “healthy”.

Clinical findings observed during physical examination and CBC or biochemical abnormalities in the 2 groups are described and compared in Tables 1, 2 and 3.

**Table 1:** Clinical findings observed at physical examination

Physical examination	SG cats (%)	CG cats (%)	Total cats (%)
No abnormalities	1 (0.7)	9 (14.3)	10 (5.1)
Lymph node enlargement^a^	121 (90.3)	38 (60.3)	159 (67.5)
Chronic gingivostomatitis^a^	50 (37.3)	12 (19)	62 (31.5)
Hyperthermia	21 (15.7)	13 (20.6)	34 (17.3)
Dehydration	17 (12.7)	5 (7.9)	22 (11.2)
BCS > 3/5^a^	1 (0.7)	16 (25.4)	17 (8.6)
BCS < 3/5	9 (6.7)	3 (4.8)	12 (6.1)
Splenomegaly	4 (3)	0	4 (2)
Hepato-splenomegaly	1(< 1)	0	1 (0.5)
Skin lesions	40 (29.8)	11 (17.5)	51 (25.8)
Ocular findings	18 (13.4)	10 (15.8)	28 (14.2)
Respiratory findings	10 (7.5)	2 (3.2)	12 (6.1)
Reproductive abnormalities	1 (0.7)	2 (3.2)	3 (1.5)
Neurologic signs	1 (0.7)	0	1 (0.5)

^a^Significant difference between SG and CG

**Table 2:** CBC abnormalities

Laboratory abnormalities	SG cats (%)	CG cats (%)	Total (%)
Anaemia^a^	53 (39.5)	15 (23.8)	68 (34.5)
Mild^a^	48 (90.6)	11 (73.3)	59 (29.9)
Moderate	5 (9.4)	2 (13.3)	7 (3.56)
Severe	–	2 (3.3)	2 (1.0)
Normocytic normochromic anaemia	43 (32.1)	11 (17.5)	54 (27.4)
Macrocytic normochromic anaemia	5 (3.7)	0 (0)	5 (2.5)
Normocytic hypochromic anaemia	2 (1.5)	1 (1.6)	3 (1.5)
Microcytic normochromic anaemia	0 (0)	3 (4.8)	3 (1.5)
Microcytic hypochromic anaemia	3 (2.2)	0 (0)	3 (1.5)
Leukocytosis^a^	46 (34.3)	5 (7.9)	51 (25.9)
Leukopenia	1 (0,7)	1 (1.6)	2 (1.0)
Neutrophilia^a^	53 (39.5)	4 (6.3)	57 (28.9)
Monocytosis^a^	38 (28.4)	9 (14.3)	47 (23.9)
Eosinophilia	12 (8.9)	6 (9.5)	18 (9.1)
Basophilia	11 (8.2)	1 (1.6)	12 (6.1)
Lymphocytosis	5 (3.7)	6 (9.5)	11 (5.6)
Lymphopenia	7 (5.2)	2 (3.2)	9 (4.6)
Neutropenia	4 (3)	0 (0)	4 (2.0)
Thrombocytopenia	5 (3.7)	2 (3.2)	7 (3.5)
Thrombocytosis	4 (3)	0 (0)	4 (2.0)

^a^Significant difference between SG and CG

**Table 3:** Biochemical abnormalities

Biochemical abnormalities	SG cats (%)	CG cats (%)	Total (%)
CK (> 320 U/l)^a^	71 (53)	18 (28.6)	89 (45.2)
AST (> 35 U/l)	36 (26.9)	16 (25.4)	52 (26.4)
ALT (> 87 U/l)	10 (7.5)	5 (7.9)	15 (7.6)
ALP (> 70 U/l)	33 (24.6)	10 (15.9)	43 (21.8)
GGT (> 0.6 U/l)	31 (23.1)	13 (20.6)	44 (22.3)
Cholinesterase (> 3950 U/l)	6 (4.5)	3 (4.8)	9 (4.6)
Total bilirubin (> 0.26 mg/dl)	6 (4.5)	4 (6.3)	10 (5.1)
Total proteins (> 7.8g/dl)	29 (21.6)	15 (23.8)	44 (22.3)
Total proteins (< 6.3g/dl)	20 (14.9)	13 (20.6)	33 (16.7)
Albumin (< 3 g/dl)^a^	88 (65.7)	19 (30.1)	107 (54.3)
Globulins (< 3 g/dl)	7 (5.2)	8 (12.7)	15 (7.6)
Globulins (> 4.5 g/dl)	47 (35.1)	14 (22.2)	61 (31)
Albumin/Globulins ratio (< 0.72)^a^	86 (64.2)	18 (28.6)	104 (52.8)
Cholesterol (> 210 mg/dl)	3 (2.2)	7 (11.1)	10 (5.1)
Cholesterol (< 95 mg/dl)	37 (27.6)	13 (20.6)	50 (25.4)
Triglycerides (> 81 mg/dl)	18 (13.4)	8 (12.7)	26 (13.2)
Triglycerides (< 19 mg/dl)	2 (1.5)	1 (1.6)	3 (1.5)
Urea (> 64 mg/dl)	27 (20.1)	14 (22.2)	41 (20.8)
Urea (< 32 mg/dl)	0	3 (4.8,7)	3 (1.5)
Creatinine (> 1.85 mg/dl)^a^	5 (3.7)	8 (12.7)	13 (6.6)
Calcium (< 11.2 g/dl)	42 (31.3)	17 (27.0)	59 (29.9)
Posphorus (> 6.6 mg/dl)^a^	74 (55.2)	20 (31.7)	94 (47.7)
Magnesium (< 81 mmol/l)	29 (21.6)	15 (23.8)	44 (22.3)
Sodium (> 152 mEq/l)^a^	32 (23.9)	39 (61.9)	71 (36)
Sodium (< 145 mEq/l)	2 (1.5)	0	2 (1.0)
Potassium (> 4.7 mEq/l)	42 (31.3)	12 (19.0)	54 (27.4)
Potassium (< 3.5 mEq/l)	1 (0.7)	3 (4.8)	4 (2.0)
Sodium/potassium ratio (< 31)^a^	31 (23.1)	6 (9.5)	37 (18.8)
Chloride (> 119 mEq/l)	18 (13.4)	16 (25.4)	34 (17.2)
Chloride (< 112 mEq/l)	5 (3.7)	1 (1.6)	6 (3.0)
Corrected Chloride (> 119 mEq/l)	87 (64.9)	40 (63.5)	127 (64.5)
Corrected Chloride (< 112 mEq/l)	2 (1.5)	0	2 (1.0)
Serum iron (> 118 μg/dl)	13 (9.7)	9 (14.3)	22 (11.2)
Serum iron (< 50 μg/dl)	28 (20.9)	11 (17.5)	39 (19.8)
UIBC (> 225 μg/dl)	42 (31.3)	20 (31.7)	62 (31.5)
UIBC (< 130 μg/dl)	10 (7.5)	6 (9.5)	16 (8.1)
TIBC (> 303 μg/dl)	39 (29.1)	23 (36.5)	62 (31.5)
TIBC (< 175 μg/dl)	5 (3,7)	3 (4.8)	8 (4.0)
Transferrin saturation (> 42.5%)	9 (6.7)	6 (9.5)	15 (7.6)
Transferrin saturation (< 19.5%)	34 (25.4)	13 (20.6)	47 (23.9)
Seric Amyloid A (> 0.5μg/ml)	42 (31.3)	14 (22.2)	56 (28.4)

^a^Significant difference between SG and CG

Skin lesions observed consisted in crusty dermatitis (*n* = 22), alopecia (*n* = 21), excoriations (*n* = 4), ulcerative dermatitis (*n* = 4), scaly dermatitis (*n* = 4), papules (*n* = 2), erythema (*n* = 2), abscess (*n* = 1) and nodule (*n* = 1). Ocular findings included corneal ulcer (*n* = 11), purulent conjunctivitis (*n* = 11), blindness (*n* =8) and retinal atrophy (*n* = 1). Respiratory findings were associated with rhinotracheitis (*n* = 11) and asthma (*n* = 1). Reproductive abnormalities included mammary hyperplasia (*n* = 1) and pyometra (*n* = 2). Neurological signs consisted in vertical nystagmus observed in one cat.

A significant difference was detected between SG and CG concerning the prevalence of three clinical findings. Lymph node enlargement (Fisher’s exact test: *P* < 0.0001, OR= 7.63, 95% CI: 3.36–17.34), and chronic gingivostomatitis (Fisher’s exact test: *P* = 0.0049, OR = 2.90, 95% CI: 1.39–6.08) were more frequent in the SG and a BCS > 3/5 (Fisher’s exact test: *P* < 0.0001, OR = 0.02, 95% CI: 0.003–0.17) was more prevalent in the CG. Moreover, anaemia (Fisher’s exact test: *P* = 0.0367, OR = 2.09, 95% CI: 1.07–4.11), mild anaemia (Fisher’s exact test: *P* = 0.0119, OR = 2.64, 95% CI: 1.26–5.53), leukocytosis (Fisher’s exact test: *P* < 0.0001, OR = 6.06, 95% CI: 2.27–16.17), neutrophilia (Fisher’s exact test: *P* < 0.0001, OR = 9.65, 95% CI: 3.31–28.15) and monocytosis (Fisher’s exact test: *P* = 0.0325, OR= 2.37, 95% CI: 1.07–5.28) were more frequently observed in SG compared to CG. Only in one case the anaemia was regenerative (1 cat of CG). Laboratory abnormalities are reported in Tables 2, 3.

Hemoparasites were not detected at the microscopical evaluation of blood smears. A significant higher prevalence of increased CK (Fisher’s exact test: *P* = 0.0013, OR = 2.82, 95% CI: 1.48–5.36), decreased albumin (Fisher’s exact test: *P* < 0.0001, OR = 4.43, 95% CI: 2.32–8.45), decreased A/G ratio (Fisher’s exact test: *P* < 0.0001, OR = 4.48, 95% CI: 2.34–8.59), increased phosphorus (Fisher’s exact test: *P* = 0.0023, OR = 2.65, 95% CI: 1.41–4.98) and decreased sodium/potassium ratio (Fisher’s exact test: *P* = 0.0303, OR = 2.86, 95% CI: 1.12–7.26) were observed in the SG when compared with CG. Conversely, increased creatinine (Fisher’s exact test: *P* = 0.0284, OR = 3.75, 95% CI: 1.17–11.99) and sodium (Fisher’s exact test: *P* < 0.0001, OR = 5.18, 95% CI: 2.72–9.88) were significantly more prevalent in the CG when compared with SG.

Urinalysis was performed in 127 cats, and 33 (25.98%) showed inappropriate (≤ 1039) urine specific gravity (21 cats in the SG and 12 in the CG). No significant difference was found between the two groups. Only two cats were proteinuric with UPC values of 0.4 in one CG cat and 2.52 in one SG cat, respectively.

### Retroviral positivity

Antibodies against FIV (15/197 = 7.6%) were detected in both groups with no significant difference. FeLV antigenemia was detected rarely (only in two cats from SG living in Calabria region). Antibodies against FIV were significantly more prevalent in adult cats compared to young (Fisher’s exact test: *P* = 0.0105, OR = 9.81, 95% CI: 1.26–76.27), and in males (12.8%) than in females (4.7%) cats (Fisher’s exact test: *P* = 0.05, OR = 2.97, 95% CI: 1.01–8.74).

### Vector-borne pathogens

#### Serological results

One hundred and seventy-three cats (87.8%) were seropositive at least to one of the tested agents and the difference between SG (91.8%), and CG (79.4%) was significant (Fisher’s exact test: *P* = 0.0187, OR = 2.91, 95% CI: 1.22–6.92). One-hundred and thirty-four cats (68%) were seropositive to two or more pathogens with a significant difference between the two groups (75.4% in SG and 60.3% in CG) (Fisher’s exact test: *P* = 0.0437, OR = 2.01, 95% CI: 1.06–3.82).

Antibody prevalence concerning the pathogens under consideration is reported in Table 4. Prevalence of anti-*R. conorii* antibodies was significantly higher in SG cats (Fisher’s exact test: *P =* 0.0094, OR = 2.30, 95% CI: 1.24–4.27)*.* High titers were detected against *B. henselae*, *R. conorii*, and *E. canis* in 10.7, 10.1 and 3.5% of tested cats, respectively, and no significant difference was found between the two groups when comparing the number of cats with high titers. Antibody prevalence to at least one pathogen was 90.8% in young cats (69/76) and 85.9% in adult cats (104/121) with no significant difference between groups.

**Table 4:** Overall antibody prevalence and titer range in the study (SG) and control (CG) groups

Antigen	Number of seroreactive cats (%)	SG positive % (titer range)	CG positive % (titer range)
*B. henselae*	90 (45.7)	49.2 (64–4096)	38 (64–2048)
R. conorii*	96 (48.7)	55.2* (64–2048)	35* (64–2048)
*E. canis*	32 (16.2)	15.7 (50–1600)	17.5 (50–1600)
*B. microti* ^a^	40 (20.3)	17.9	25.4
*A. phagocytophilum*	53 (26.9)	29.1 (50–400)	22.2 (50–400)
*L. infantum*	19 (9.6)	7.5 (80–160)	11.1 (80–320)

^*^Significant difference of prevalence between SG and CG

^a^Titer not determined

Moreover, 87.2% of Sicilian cats (100% from SG and 73.7% from CG) and 90.5% of Calabrian cats (93% in SG and 84% in CG) were positive at least to one VBP. Antibody prevalence for *R. conorii* (Fisher’s exact test: *P* = 0.0062, OR = 5.24, 95% CI: 1.45–18.97) and *B. microti* (Fisher’s exact test: *P* = 0.0459, OR = 3.36, 95% CI: 1.16, 9.76) were significantly higher in the SG cats from Sicily compared to those from Calabria. No other significant difference was found between the two regions.

#### Molecular assays

Positive PCR tests were obtained for *Bartonella* spp. (21.3%), *Mycoplasma* spp. (18.3%) and *L. infantum* (6.6%) (Table 5) but they were negative for *Ehrlichia/Anaplasma* spp., piroplasmids (*Babesia* spp. and *Theileria* spp.), *Rickettsia* spp*.* and *Hepatozoon felis.* The following species were sequenced (Table 5): *B. henselae*, *B. clarridgeiae*, *M. haemofelis*, “*Ca.* Mycoplasma haemominutum,” “*Ca.* Mycoplasma turicensis” and *L. infantum*.

**Table 5:** Positive PCR and sequencing results

Pathogen	Number of positive cats (%)		
	Total cats (*n* = 197)	SG cats (*n* = 134)	CG cats (*n* = 63)
*Bartonella* spp.^a^	42 (21.3)	39 (29.1)	3 (4.8)
*B. henselae*	30 (15.2)	28 (20.9)	2 (3.2)
*B. clarridgeiae*	12 (6.1)	11 (8.2)	1 (1.6)
*Mycoplasma* spp.	36 (18.3)	27 (20.2)	9 (14.3)
*Mycoplasma haemofelis*	12 (6.1)	11 (8.2)	1 (1.6)
“*Candidatus* Mycoplasma haemominutum”	26 (13.2)	18 (13.4)	8 (12.7)
“*Candidatus* Mycoplasma turicensis”	10 (5.1)	8 (6.0)	2 (3.2)
*Leishmania infantum*	13 (6.6)	8 (6.0)	5 (7.9)
Conjunctival swabs (*n* = 394)	3	2	1
Oral swabs (*n* = 197)	3	0	3
Blood EDTA (*n* = 197)	4	4	0
Lymph nodes (*n* = 181)	3	2	1
Urine samples (*n* = 143)	3	2	1
Total	79 (35)	63 (47)	16 (25.4)

^a^Significant difference between SG and CG

*Bartonella* spp. prevalence was significantly higher in SG compared to CG (Fisher’s exact test: *P* < 0.0001, OR= 8.21, 95% CI: 2.43–27.76). *Mycoplasma* spp. infection was significantly more frequent in FIV positive cats compared to FIV negative cats (Fisher’s exact test: *P* = 0.0002, OR = 8.61, 95% CI: 2.83–26.16). Overall, 79 cats (40.1%) were PCR positive at least to one of the tested agents and this category was significantly more prevalent in the SG (47%) compared to the CG (25.4%) (Fisher’s exact test: *P* = 0.0049, OR = 2.61, 95% CI: 1.35–5.05). There was no significant difference for molecular positivity rate to at least one pathogen according to gender, age and region.

*Leishmania infantum* DNA was amplified in some cases from two specimens/cat as follows: blood and lymph node (*n* = 2), blood and urine (*n* = 1) or conjunctival and oral swabs (*n* = 1). Parasitic load for *L. infantum* ranged from 1 to 80,000 *Leishmania*/ml detected in blood EDTA, from 1 to 11,000 *Leishmania*/specimen in lymph nodes, from 7 to 120 *Leishmania*/specimen in conjunctival swabs, from 16 to 92 *Leishmania*/specimen in oral swabs, and from 1 to 30 *Leishmania*/ml in urine.

Co-infections with at least two pathogens were found in 10.1% of cats with 13.4% of SG cats (*n* = 18) and 3.2% of CG cat (*n* = 2). Only 2 cats were co-infected with three pathogens: one (1.6%) from CG (*L. infantum*, *M. haemofelis* and “*Ca.* Mycoplasma haemominutum”) and one (0.7%) form SG (*B. henselae*, *M. haemofelis* and “*Ca.* Mycoplasma haemominutum”). The most common co-infection detected was between *Bartonella* spp. and *Mycoplasma* spp. in six cats. Co-infections with *L. infantum* and other pathogens included two with “*Ca.* Mycoplasma haemominutum”, two with *B. henselae* and one with *B. clarridgeiae*. Co-infections with different *Mycoplasma* species included “*Ca.* Mycoplasma haemominutum” and “*Ca.* Mycoplasma turicensis” (5 cats), *M. haemofelis* and “*Ca.* Mycoplasma haemominutum” (3 cats), and “*Ca.* Mycoplasma haemominutum”, “*Ca.* Mycoplasma turicensis” and *M. haemofelis* (2 cats).

#### Overall exposure prevalence

An overall prevalence of exposure was calculated for pathogens investigated by both serological and molecular methods. Overall, *Bartonella* spp. exposure was 48.7% (96/197) and a higher prevalence was observed in FIV positive cats (Fisher’s exact test: *P* = 0.0002, OR = 17.07, 95% CI: 2.20–132.65). Overall, *L. infantum* positivity rate was 14.7% (29/197), but no significant difference was observed between SG and CG or according to age, gender or FIV positivity.

One-hundred-eighty-two cats (92.4%) were found positive (by IFAT and PCR) to at least one VBP. The SG cats had higher (96.3%) exposure prevalence to at least one VBP compared to CG (84.1%) (Fisher’s exact test: *P* = 0.0068, OR = 4.87, 95% CI: 1.59–14.93).

Two or more co-infections were detected in 62 cats (31.5%). In SG, there was a higher prevalence (37.3%) of multiple positivity compared to CG (19%) (Fisher’s exact test: *P* = 0.0132, OR = 2.53, 95% CI: 1.23–5.20]). Conversely, no difference was found in FIV positive cats compared to FIV negative cats.

#### Multivariable logistic regression analysis

Seventeen variables that showed a significative difference in prevalence of one or more VBP between SG and CG with univariate analysis entered the multivariable logistic regression analysis performed on the 197 cats. Significant associations were found for exposure to *L. infantum*, *Bartonella* spp., *Mycoplasma* spp., *B. microti*, and *A. phagocytophilum* (Table 6) and co-infections (Additional file 3: Table S3). The significant associations concerned adult age and hemoplasma infection, FIV positivity and *Bartonella* spp. or hemoplasma infection, anaemia and hemoplasma infection, elevated ALP serum activity or low albumin concentration and *A. phagocytophilum* antibody positivity, high globulin concentration and *Bartonella* spp. infection, and high creatinine concentration with hemoplasma infection. Moreover, adults had a lower risk of *B. microti* antibody positivity compared to young cats; elevated serum CK activity was less likely in cats with *L. infantum*, hemoplasma, co-infections, or *B. microti* antibody positivity; cats with *Bartonella* spp infection were less likely to display elevated ALP serum activity.

**Table 6:** Multivariable logistic regression analysis of VBP

Variables with significant ORs	*Leishmania infantum* ^a^		*Bartonella* spp.^a^		*Mycoplasma* spp.^b^		*Babesia microti* ^c^		*Anaplasma phagocytophilum* ^c^	
	OR (95% Cl)	*P*	OR (95% Cl)	*P*	OR (95% Cl)	*P*	OR (95% Cl)	*P*	OR (95% Cl)	*P*
Adult age		ns		ns	4.53 (1.23–16.74)	0.02	0.28 (0.11–0.75)	< 0.01		ns
FIV positivity		ns	11.79 (1.38–100.97)	0.02	5.07 (1.33–19.33)	0.02		ns		ns
Anaemia		ns		ns	2.76 (1.05–7.29)	0.04		ns		ns
High CK	0.28 [0.10; 0.78]	0.01		ns	0.35 (0.12–0.98)	0.04	0.31 (0.12–0.69)	0.01		ns
High ALP		ns	0.26 (0.09–0.71)	0.01		ns		ns	4.40 (1.54–12.57)	< 0.001
Low Albumin		ns		ns		ns		ns	2.42 (1.05–5.59)	0.04
High Globulins		ns	2.99 (1.38–6.48)	0.00		ns		ns		ns
High Creatinine		ns		ns	7.68 (1.43–41.01)	0.02		ns		ns

*Abbreviation*: *ns* not significant

^a^PCR and/or IFAT

^b^PCR

^c^IFAT

## Discussion

This controlled field study examined the prevalence of selected VBP and the clinical and clinicopathological abnormalities in cats exposed to ectoparasites. Moreover, some risk factors for VBP exposure were identified using significant associations detected by multivariable logistic regression analysis between some of the investigated pathogens and independent variables.

The study was based on both antibody and molecular detection of feline VBPs under consideration to increase the possibility of evaluating exposure of cats to tested pathogens. We found that cats were extremely exposed to VBP because of the high antibody (88.3%), molecular (40%), and overall (antibody and PCR) positivity (92.4%) to at least one pathogen. Moreover, cats exposed to ectoparasites, because of their outdoor lifestyle and the lack of regular ectoparasiticide treatments (SG), showed a significantly higher molecular and overall (antibody and PCR) positivity compared to indoor cats with no ectoparasites and subjected to regular application of ectoparasiticides (CG). Exposure to multiple VBP was also very frequent, as about two-thirds of tested cats were antibody positive, 10% PCR positive and 30.1% antibody and PCR positive to more than one VBP at the time of sampling; the difference between SG and CG was significant for antibody or antibody and molecular positivity. Epidemiological similarities shared by some VBP can obviously be responsible for vector-borne co-infections but also other factors, such as pathogenic interactions between them, can delay or prevent clearance of VBI and concur to co-infections. Other studies detected a high overall blood PCR positivity to at least one VBP, i.e. 25–29.9% in Portugal [4, 17], 48.9% in northern Italy [18] and 45.4% in Cyprus [3]. Prevalence of co-infections was lower (2.2–9.8%) in those studies [3, 4, 17, 18].

A small percentage of enrolled cats (12%) were antibody negative to all of VBP, and this percentage was significantly higher in the CG. However, we found a significant percentage of CG cats positive to the same VBP detected in the SG, and this may be explained by lack of compliance to ectoparasiticide treatment, low efficacy of the used ectoparasiticide, and/or non-vectorial transmission of the pathogen. For instance, in the case of *L. infantum* infection, pyrethroids are used in dogs for sand fly bite prevention, but almost all of these compounds are toxic to cats, and only a collar containing flumethrin and imidacloprid was able to reduce the incidence of *L. infantum* infection in cats [19–21]. Blood transfusion is a main non-vectorial transmission route of feline VBI but, at least for hemoplasmas, other routes are strongly suspected [22–24]. Moreover, our model of multivariable analysis did not find significant associations between exposure to any individual or multiple VBP and outdoor lifestyle or individual ectoparasiticide treatment. Attipa et al. [3] also used multivariable logistic regression to investigate risk factors of some VBPs detected in cats from Cyprus and, similarly, they did not find any association between positivity to any tested VBP and the lack of ectoparasiticide use while they found an association of outdoor lifestyle only with hemoplasma positivity [3].

Based on antibody detection, *R. conorii* (or other cross-reacting *Rickettsia* species) was the most frequent agent circulating among tested cats (48.7%), and it was significantly more common in cats exposed to ectoparasites (SG). *Rickettsia conorii* is historically the most important zoonotic species of the *Rickettsia* genus in the Mediterranean area, and was recently confirmed as a possible causative agent of acute febrile illness in dogs showing a transient positive blood PCR and seroconversion [25]. Studies about the infection of cats with *Rickettsia* spp. of the Mediterranean spotted fever group reported the collection of infected ticks on cats, and obtained positive blood PCR and a high antibody prevalence [10, 17, 26–28]. These data support the need for prospective investigations about the pathogenic role of spotted fever group *Rickettsia* spp. in cats.

Antibody prevalence for *B. henselae* was high (45.7%), as was the seroreactivity for *A. phagocytophilum* (26.9%), *B. microti* (20.3%) and *E. canis* (16.2%) antigens. In many previous serological studies performed by IFAT, antibody titer against tested VBP was not reported, or they were low [6, 10, 29, 30]. Interestingly, we detected high titers against *B. henselae* (10.7%), *R. conorii* (10.1%) and *E. canis* (3.5%) antigens; however, we cannot exclude the possibility of serological cross-reactions with other species of the same genus. This is most important for *R. conorii* and *E. canis* because we documented the exposure of cats by serology, but we were not able to find the pathogen DNA in blood as shown in other studies [31, 32]. Similarly, we did not detect DNA of *Anaplasma*, *Hepatozoon* and piroplasmids such as *Babesia* or *Cytauxzoon*. This might be a consequence of the lack of exposure, clearance or sequestration of the organisms in other tissues, or technical limitations. However, in a study performed on blood collected from outdoor cats from a confined area of Sicily, only *Hepatozoon felis* DNA was detected in one cat (0.3%). Therefore, it is likely that some VBPs are not common in Sicily and the Calabria region [33]. *Anaplasma*/*Ehrlichia* DNA detection is rare in cats, and more frequently *A. phagocytophilum* is amplified [4, 17, 31, 34], but *A. platys* or *A. platys*-like organisms were also occasionally characterised in cats in southern Europe and the seropositivity detected in this study could be due to different species of *Anaplasma* [3, 35]. However, *Ehrlichia* spp. and *Anaplasma* spp. were not found in other studies in Greece and Spain [36, 37].

*Hepatozoon felis* distribution seems to be quite variable in southern Europe. In fact, it is very rare in Italy, but a focus was recently detected in Matera, where single cases of *Hepatozoon canis* and *Hepatozoon silvestris* infections were also seen [10, 33, 38]. *Hepatozoon felis* is reported in the Iberian Peninsula with a prevalence range of 1.6–13.8% [4, 17, 31, 39] and the highest molecular prevalence (37.9%) was recently found in Cyprus [3].

Feline piroplasmid infection is uncommon in Europe. Piroplasmid infection is caused by *Cytauxzoon* spp. [39–43], *Babesia vogeli*, or *Babesia canis* [4, 17]. In Italy, DNA of *B. microti* was sequenced in cats from Sicily and Milan but, despite 20.3% antibody prevalence for *B. microti* obtained in this study, we were not able to detect piroplasmid DNA, and we cannot exclude that other *Babesia* spp. elicited antibody production in cats [44, 45]. Interestingly, adult cats were less likely to have antibodies to *B. microti* compared to those < 1 year of age. Young cats might therefore be more susceptible to *Babesia* spp. infection than adults but the antibody response would not persist possibly because of clearance of the infection.

*Bartonella* spp. was VBP with the highest overall (48.7%) and molecular (21.3%) prevalence in this study, and molecular prevalence was significantly higher in SG. A significant association between *Bartonella* spp. exposure and FIV infections or increased globulin values were found by multivariate analysis as previously reported by univariate analysis only for globulins [46] but not for FIV [3, 5]. A very high *B. henselae* antibody prevalence (45.7%) was also found. Two species were sequenced, and *B. henselae* was more prevalent (15.2%) than *B. clarridgeiae* (6.1%). These results confirm data obtained in 42 cats from the same area carrying ticks or fleas at the time of examination, where blood PCR positivity for *Bartonella* spp. was 38.1%, and *B. henselae* (21.4%) was more frequently detected than *B. clarridgeiae* (16.6%) [10]. A higher molecular prevalence was previously found in Sicily in a study using a nested-PCR where positivity was 70.6% in blood, 72.9% in lymph node aspirates, and 60.0% in oral swabs and *B. henselae* was the only sequenced species [8]. In other European countries, lower blood PCR prevalences were reported ranging 1–22.4%, and this might be due to the exposure of investigated cats to fleas or the assays used [3–5, 28, 31, 36, 37]. However, also in these latter studies, *B. henselae* was usually more frequent than *B. clarridgeiae* and only in one cat was *B. kholerae* DNA was sequenced [37].

As in other studies performed in Italy [10, 18], Cyprus [3], and Portugal [16, 47], we frequently detected hemoplasma DNA in cat blood (18.3%) and “*Ca.* Mycoplasma haemominutum” was more commonly sequenced, compared to “*Ca.* Mycoplasma turicensis” and *M. haemofelis*. Other studies reported lower prevalence (range 7.8–14.9%) but similar species were sequenced and represented possible co-infections [12, 36, 48, 49]. We obtained significant associations by multivariate analysis between hemoplasma positivity and anaemia (OR= 2.76), adult age (OR= 4.53), FIV positivity (OR= 5.07), and increased creatinine values (OR=7.68). Interestingly, hemoplasma positive cats have seven times higher risk for an increased creatinine concentration and this association was never found before by multivariate analysis. We performed a cross-sectional study and therefore did not have the possibility to confirm chronic kidney disease (CKD) in cats with high creatinine; however, in two-thirds of cats urinalysis showed inappropriate urine specific gravity that is suggestive of CKD. A causative role for variables cannot be assessed by cross-sectional investigations, and prospective surveys on hemoplasma long carrier cats should be considered. Older age of hemoplasma-positive cats was also found in two other studies using multivariable logistic analysis, and could contribute to the association between hemoplasma positivity and high creatinine concentrations [3, 49]. Hemolytic anaemia is the main pathogenic effect of *M. haemofelis* and less frequently of other hemoplasmas, but subclinical carriers can be found, and this may explain when in other studies multivariate analysis did not find associations between hemoplasmas and anaemia [3, 49]. Conversely, significant association with FIV was previously reported by other authors using multivariable analysis, and this comorbidity could be due to epidemiological factors (sharing of the way of transmission) or facilitation of long-term hemoplasma infections in FIV positive cats [3, 49, 50].

Feline *L. infantum* infection can be considered an emergent VBI in endemic areas of canine leishmaniosis [20]. Many studies evaluated antibody and or molecular prevalence in southern Italy, and a wide prevalence range was found by both antibody detection (2.4–59%) and blood PCR (7.1–61%) [10, 33, 51–53]. Epidemiological (endemicity, characteristics of the studied population) and technical (serological cut-off, molecular technique) differences may account for this variability. Antibody prevalence obtained in this study was within the above range (9.6%), but blood DNA detection was as low as 2.0%. Low molecular prevalence was also found by non-invasive samplings as conjunctival (1.5%) and oral (1.5%) swabs, or by lymph node aspirate (1.7%) or urine (2%). Interestingly, high parasite loads were obtained only from blood (up to 80,000 *Leishmania*/ml), and the clinical relevance of this finding lies in the risk of iatrogenic transmission of *L. infantum* by blood transfusion as reported in dogs [54, 55].

Co-infection of *L. infantum* with “*Ca.* Mycoplasma haemominutum”*, B. henselae* or *B. clarridgeiae* was similar to that reported in other studies [3, 10]. However, other *L. infantum* co-infections are known including *Anaplasma/Ehrlichia* spp., *Babesia* spp., *Hepatozoon* spp., and *Borrelia burgdorferi* [3, 4, 17].

Finally, the univariate analysis pointed out some clinical and clinicopathological abnormalities that were significantly more frequent in outdoor cats exposed to ectoparasites than in indoor cats protected from ectoparasites despite the former being significantly younger and less frequently admitted for health problems. Other infectious and parasite agents in addition to VBP frequently affect outdoor cats and may be responsible for this occurrence [28, 36, 56–58]. The only biochemical abnormality more frequent in indoor cats was increased creatinine, but these cats were significantly older than those of SG, and this bias could influence the result. Indoor cats were also significantly more overweight compared to outdoor cats and therefore are predisposed to metabolic or urinary problems [59–61]. The clinical relevance of these findings is that lifestyle significantly influences the health of cats and adequate preventative measures have to be tailored accordingly.

## Conclusions

A very high prevalence of zoonotic VBP exposure was found in cats, with *Rickettsia* spp. and *Bartonella* spp. being the most prevalent. Overall, cats exposed to ectoparasites for the lack of preventative measure and/or an outdoor lifestyle had a higher risk for VBI and co-infections and for some clinical and clinicopathological abnormalities. Some risk factors were documented by multivariable logistic regression analysis providing a better understanding of the epidemiology for selected feline pathogens, namely for *Mycoplasma* spp. and *Bartonella* spp. infections that were both found associated with FIV.

Also, the lifestyle of cats appeared clinically relevant for diseases other than those associated with VBP exposure and requires specific preventative measures to protect their health.

## Additional files

Additional file 1: **Table S1**. Feline CBC reference intervals. (DOCX 15 kb)

Additional file 2: **Table S2**. Serum biochemistry reference intervals. (DOCX 15 kb)

Additional file 3: **Table S3.** Ordered logistic regression analysis of VBP. (DOCX 14 kb)

## Abbreviations

ALP: alkaline phosphatase
ALT: alanine aminotransferase
AST: aspartate aminotransferase
BCS: body condition score
CBC: complete blood count
CG: control group
CI: confidence interval
CK: creatine kinase
CKD: chronic kidney disease
GGT: gamma glutamyl transferase
OR: odds ratio
SAA: serum amyloid A
SG: study group
TIBC: total iron-binding capacity
UIBC: unsatured iron-binding capacity
VBI: vector-borne infections
VBP: vector-borne pathogen

## References

[1] Otranto D, Dantas-Torres F (2010). Canine and feline vector-borne diseases in Italy: current situation and perspectives. Parasit Vectors..

[2] Scorza AV, Lappin MR. Prevalence of selected zoonotic and vector-borne agents in dogs and cats on the Pine Ridge Reservation. Vet Sci. 2017;4 10.3390/vetsci4030043 PMC5644658 29056701

[3] Attipa C, Papasouliotis K, Solano-Gallego L, Baneth G, Nachum-Biala Y, Sarvani E (2017). Prevalence study and risk factor analysis of selected bacterial, protozoal and viral, including vector-borne, pathogens in cats from Cyprus. Parasit Vectors..

[4] Maia C, Ramos C, Coimbra M, Bastos F, Martins A, Pinto P (2014). Bacterial and protozoal agents of feline vector-borne diseases in domestic and stray cats from southern Portugal. Parasit Vectors..

[5] Bergmann M, Hartmann K (2017). Vector-borne diseases in cats in Germany. Tierarztl Prax Ausg K Klientiere Heimtiere..

[6] Ayllón T, Diniz PPVP, Breitschwerdt EB, Villaescusa A, Rodríguez-Franco F, Sainz A (2012). Vector-borne diseases in client-owned and stray cats from Madrid, Spain. Vector Borne Zoonotic Dis..

[7] Solano-Gallego L, Rossi L, Scroccaro AM, Montarsi F, Caldin M, Furlanello T (2012). Detection of *Leishmania infantum* DNA mainly in *Rhipicephalus sanguineus* male ticks removed from dogs living in endemic areas of canine leishmaniosis. Parasit Vectors..

[8] Pennisi MG, La Camera E, Giacobbe L, Orlandella BM, Lentini V, Zummo S (2010). Molecular detection of *Bartonella henselae* and *Bartonella clarridgeiae* in clinical samples of pet cats from Southern Italy. Res Vet Sci..

[9] Pennisi MG (2010). La leishmaniosis felina dalla A alla Z. Proceedings 2nd International Congress on Canine Leishmaniasis.

[10] Persichetti MF, Solano-Gallego L, Serrano L, Altet L, Reale S, Masucci M (2016). Detection of vector-borne pathogens in cats and their ectoparasites in southern Italy. Parasit Vectors..

[11] Pennisi MG, Persichetti MF, Serrano L, Altet L, Reale S, Gulotta L (2015). Ticks and associated pathogens collected from cats in Sicily and Calabria (Italy). Parasit Vectors..

[12] Bergmann M, Englert T, Stuetzer B, Hawley JR, Lappin MR, Hartmann K (2017). Risk factors of different hemoplasma species infections in cats. BMC Vet Res..

[13] Piaton E, Fabre M, Goubin-Versini I, Bretz-Grenier M-F, Courtade-Saïdi M, Vincent S (2016). Guidelines for May-Grünwald-Giemsa staining in haematology and non-gynaecological cytopathology: recommendations of the French Society of Clinical Cytology (SFCC) and of the French Association for Quality Assurance in Anatomic and Cytologic Pathology (AFAQAP). Cytopathol Off J Br Soc Clin Cytol..

[14] International Renal Interest Society. IRIS Staging of CKD. www.iris-kidney.com/pdf/003-5559.001-iris-website-staging-of-ckd-pdf_220116-final.pdf#page=7. Accessed 22 Dec 2017.

[15] Persichetti MF, Solano-Gallego L, Vullo A, Masucci M, Marty P, Delaunay P (2017). Diagnostic performance of ELISA, IFAT and Western blot for the detection of anti-*Leishmania infantum* antibodies in cats using a Bayesian analysis without a gold standard. Parasit Vectors..

[16] Martínez-Díaz VL, Silvestre-Ferreira AC, Vilhena H, Pastor J, Francino O, Altet L (2013). Prevalence and co-infection of haemotropic mycoplasmas in Portuguese cats by real-time polymerase chain reaction. J Feline Med Surg..

[17] Vilhena H, Martinez-Díaz VL, Cardoso L, Vieira L, Altet L, Francino O (2013). Feline vector-borne pathogens in the north and centre of Portugal. Parasit Vectors..

[18] Spada E, Proverbio D, Galluzzo P, Della Pepa A, Perego R, Bagnagatti De Giorgi G (2014). Molecular study on selected vector-borne infections in urban stray colony cats in northern Italy. J Feline Med Surg..

[19] Solano-Gallego L, Miró G, Koutinas A, Cardoso L, Pennisi MG, Ferrer L (2011). LeishVet guidelines for the practical management of canine leishmaniosis. Parasit Vectors..

[20] Pennisi MG, Cardoso L, Baneth G, Bourdeau P, Koutinas A, Miró G (2015). LeishVet update and recommendations on feline leishmaniosis. Parasit Vectors..

[21] Brianti E, Falsone L, Napoli E, Gaglio G, Giannetto S, Pennisi MG (2017). Prevention of feline leishmaniosis with an imidacloprid 10%/flumethrin 4.5% polymer matrix collar. Parasit Vectors..

[22] Museux K, Boretti FS, Willi B, Riond B, Hoelzle K, Hoelzle LE (2009). *In vivo* transmission studies of “*Candidatus* Mycoplasma turicensis” in the domestic cat. Vet Res..

[23] Pennisi MG, Hartmann K, Addie DD, Lutz H, Gruffydd-Jones T, Boucraut-Baralon C (2015). Blood transfusion in cats: ABCD guidelines for minimising risks of infectious iatrogenic complications. J Feline Med Surg..

[24] Lappin MR. Feline haemoplasmas are not transmitted by *Ctenocephalides felis*. Abstr Book, Lisbon, Portugal: CVBD World. Forum. 2014:44–6.

[25] Solano-Gallego L, Caprì A, Pennisi MG, Caldin M, Furlanello T, Trotta M (2015). Acute febrile illness is associated with *Rickettsia* spp infection in dogs. Parasit Vectors..

[26] Solano-Gallego L, Hegarty B, Espada Y, Llull J, Breitschwerdt E (2006). Serological and molecular evidence of exposure to arthropod-borne organisms in cats from northeastern Spain. Vet Microbiol..

[27] Segura F, Pons I, Miret J, Pla J, Ortuño A, Nogueras M-M (2014). The role of cats in the eco-epidemiology of spotted fever group diseases. Parasit Vectors..

[28] Diakou A, Di Cesare A, Accettura PM, Barros L, Iorio R, Paoletti B (2017). Intestinal parasites and vector-borne pathogens in stray and free-roaming cats living in continental and insular Greece. PLoS Negl Trop Dis..

[29] Ayllón T, Villaescusa A, Tesouro MA, Sainz A. Serology, PCR and culture of *Ehrlichia/Anaplasma* species in asymptomatic and symptomatic cats from central Spain. Clin Microbiol Infect. 2009;15(Suppl. 2):4-5. 10.1111/j.1469-0691.2008.02645.x 19374640

[30] Ebani VV, Bertelloni F, Fratini F (2012). Occurrence of *Bartonella henselae* types I and II in Central Italian domestic cats. Res Vet Sci..

[31] Tabar M-D, Altet L, Francino O, Sánchez A, Ferrer L, Roura X (2008). Vector-borne infections in cats: molecular study in Barcelona area (Spain). Vet Parasitol..

[32] Bayliss DB, Morris AK, Horta MC, Labruna MB, Radecki SV, Hawley JR (2009). Prevalence of *Rickettsia* species antibodies and *Rickettsia* species DNA in the blood of cats with and without fever. J Feline Med Surg..

[33] Otranto D, Napoli E, Latrofa MS, Annoscia G, Tarallo VD, Greco G (2017). Feline and canine leishmaniosis and other vector-borne diseases in the Aeolian Islands: pathogen and vector circulation in a confined environment. Vet Parasitol..

[34] Bergmann M, Englert T, Stuetzer B, Hawley JR, Lappin MR, Hartmann K (2015). Prevalence of selected rickettsial infections in cats in southern Germany. Comp Immunol Microbiol Infect Dis..

[35] Zobba R, Anfossi AG, Visco S, Sotgiu F, Dedola C, Pinna Parpaglia ML (2015). Cell tropism and molecular epidemiology of *Anaplasma platys*-like strains in cats. Ticks Tick-Borne Dis..

[36] Ravicini S, Pastor J, Hawley J, Brewer M, Castro-López J, Beall M (2016). Prevalence of selected infectious disease agents in stray cats in Catalonia, Spain. JFMS Open Rep.

[37] Mylonakis ME, Schreeg M, Chatzis MK, Pearce J, Marr HS, Saridomichelakis MN, et al. Molecular detection of vector-borne pathogens in Greek cats. Ticks Tick-Borne Dis. 2017; 10.1016/j.ttbdis.2017.08.013 28887102

[38] Giannelli A, Latrofa MS, Nachum-Biala Y, Hodžić A, Greco G, Attanasi A (2017). Three different *Hepatozoon* species in domestic cats from southern Italy. Ticks Tick-Borne Dis..

[39] Díaz-Regañón D, Villaescusa A, Ayllón T, Rodríguez-Franco F, Baneth G, Calleja-Bueno L (2017). Molecular detection of *Hepatozoon* spp. and *Cytauxzoon* sp. in domestic and stray cats from Madrid, Spain. Parasit Vectors..

[40] Carli E, Trotta M, Chinelli R, Drigo M, Sinigoi L, Tosolini P (2012). *Cytauxzoon* sp. infection in the first endemic focus described in domestic cats in Europe. Vet Parasitol..

[41] Carli E, Trotta M, Bianchi E, Furlanello T, Caldin M, Pietrobelli M (2014). *Cytauxzoon* sp. infection in two free-ranging young cats: clinicopathological findings, therapy and follow up. Turk Parazitolojii Derg..

[42] Alho AM, Silva J, Fonseca MJ, Santos F, Nunes C, de Carvalho LM (2016). First report of *Cytauxzoon* sp. infection in a domestic cat from Portugal. Parasit Vectors..

[43] Legroux J-P, Halos L, René-Martellet M, Servonnet M, Pingret J-L, Bourdoiseau G (2017). First clinical case report of *Cytauxzoon* sp. infection in a domestic cat in France. BMC Vet Res..

[44] Pennisi MG, Alongi A, Agnone A, Vitale F, Reale S, Torina A (2007). Cats as reservoir of *Babesia microti*. Parassitologia.

[45] Spada E, Proverbio D, Galluzzo P, Perego R, Bagnagatti De Giorgi G, Roggero N (2014). Frequency of piroplasms *Babesia microti* and *Cytauxzoon felis* in stray cats from northern Italy. BioMed Res Int..

[46] Whittemore JC, Hawley JR, Radecki SV, Steinberg JD, Lappin MR (2012). *Bartonella* species antibodies and hyperglobulinemia in privately owned cats. J Vet Intern Med..

[47] Duarte A, Marques V, Correia JHD, Neto I, Bráz BS, Rodrigues C (2015). Molecular detection of haemotropic *Mycoplasma* species in urban and rural cats from Portugal. J Feline Med Surg..

[48] Rosenqvist MB, Meilstrup A-KH, Larsen J, Olsen JE, Jensen AL, Thomsen LE (2016). Prevalence of feline haemoplasma in cats in Denmark. Acta Vet Scand..

[49] Ravagnan S, Carli E, Piseddu E, Da Rold G, Porcellato E, Zanardello C (2017). Prevalence and molecular characterization of canine and feline hemotropic mycoplasmas (hemoplasmas) in northern Italy. Parasit Vectors..

[50] Jenkins KS, Dittmer KE, Marshall JC, Tasker S (2013). Prevalence and risk factor analysis of feline haemoplasma infection in New Zealand domestic cats using a real-time PCR assay. J Feline Med Surg..

[51] Pennisi MG, Masucci M, Catarsini O (1998). Presenza di anticorpi anti-*Leishmania* in gatti FIV+ che vivono in zona endemica. Società Italiana delle Scienze Veterinarie - Atti 52° Convegno.

[52] Pennisi MG, Maxia L, Vitale F, Masucci M, Borruto G, Caracappa S (2000). Studio sull’infezione da *Leishmania* mediante PCR in gatti che vivono in zona endemica. Società Italiana delle Scienze Veterinarie - Atti 54° Convegno.

[53] Pennisi MG, Lupo T, Malara D, Masucci M, Migliazzo A, Lombardo G. Serological and molecular prevalence of *Leishmania infantum* infection in cats from southern Italy. J Feline Med Surg. 2012;14:656–7.

[54] de Freitas E, Melo MN, da Costa-Val AP, Michalick MSM (2006). Transmission of *Leishmania infantum via* blood transfusion in dogs: potential for infection and importance of clinical factors. Vet Parasitol..

[55] Owens SD, Oakley DA, Marryott K, Hatchett W, Walton R, Nolan TJ (2001). Transmission of visceral leishmaniasis through blood transfusions from infected English foxhounds to anemic dogs. J Am Vet Med Assoc..

[56] Diakou A, Di Cesare A, Barros LA, Morelli S, Halos L, Beugnet F (2015). Occurrence of *Aelurostrongylus abstrusus* and *Troglostrongylus brevior* in domestic cats in Greece. Parasit Vectors..

[57] Piyarungsri K, Pusoonthornthum R (2017). Risk and protective factors for cats with naturally occurring chronic kidney disease. J Feline Med Surg..

[58] Hwang J, Gottdenker N, Min M-S, Lee H, Chun M-S (2016). Evaluation of biochemical and haematological parameters and prevalence of selected pathogens in feral cats from urban and rural habitats in South Korea. J Feline Med Surg..

[59] Lund HS, Sævik BK, Finstad ØW, Grøntvedt ET, Vatne T, Eggertsdóttir AV (2016). Risk factors for idiopathic cystitis in Norwegian cats: a matched case-control study. J Feline Med Surg..

[60] Rowe E, Browne W, Casey R, Gruffydd-Jones T, Murray J (2015). Risk factors identified for owner-reported feline obesity at around one year of age: dry diet and indoor lifestyle. Prev Vet Med..

[61] Chandler M, Cunningham S, Lund EM, Khanna C, Naramore R, Patel A (2017). Obesity and associated comorbidities in people and companion animals: a One Health perspective. J Comp Pathol..

