# Supplementary material for: Clinical evaluation of outdoor cats exposed to ectoparasites and associated risk for vector-borne infections in southern Italy
Source: Parasit Vectors. 2018 Mar 20;11:136. doi: 10.1186/s13071-018-2725-8 (PMC5859451; doi:10.1186/s13071-018-2725-8)
Supplement: Supplementary file 1 — Table S1. Feline CBC reference intervals. (DOCX 15 kb) [file 13071_2018_2725_MOESM1_ESM.docx]

**Additional file 1: Table S1**. Feline CBC reference intervals

| **Parameter** | **Reference interval** | |
| --- | --- | --- |
|  | **Min** | **Max** |
| Red Blood Cells (10^6^/µl) | 6.54 | 12.20 |
| Hematocrit (%) | 30.3 | 52.3 |
| Hemoglobin (g/dl) | 9.8 | 16.2 |
| Mean Corpuscular Volume (fl) | 35.9 | 53.1 |
| Mean Corpuscular Hemoglobin (pg) | 11.8 | 17.3 |
| Mean Corpuscular Hemoglobin Concentration (g/dl) | 28.1 | 35.8 |
| Red Cell Distribution Width (%) | 15.0 | 27.0 |
| Reticulocytes (10^3^/µl) | 3.0 | 50.0 |
| White Blood Cells (10^3^/µl) | 2.87 | 17.02 |
| Neutrophils (10^3^/µl) | 1.48 | 10.29 |
| Lymphocytes (10^3^/µl) | 0.92 | 6.88 |
| Monocytes (10^3^/µl) | 0.05 | 0.67 |
| Eosinophils (10^3^/µl) | 0.17 | 1.57 |
| Basophils (10^3^/µl) | 0.01 | 0.26 |
| Platelets (10^3^/µl) | 151 | 600 |
| **Degree of anemia** | **Hematocrit (%)** | |
| mild | 20 | 30.2 |
| moderate | 14 | 19 |
| severe | 0 | 13 |
