# Supplementary material for: Clinical evaluation of outdoor cats exposed to ectoparasites and associated risk for vector-borne infections in southern Italy
Source: Parasit Vectors. 2018 Mar 20;11:136. doi: 10.1186/s13071-018-2725-8 (PMC5859451; doi:10.1186/s13071-018-2725-8)
Supplement: Supplementary file 2 — Table S2. Serum biochemistry reference intervals. (DOCX 15 kb) [file 13071_2018_2725_MOESM2_ESM.docx]

**Additional file 2: Table S2**. Serum biochemistry reference intervals

| **Parameter** | **Reference interval** | |
| --- | --- | --- |
|  | **Min** | **Max** |
| CK (UI/l) | 90 | 320 |
| AST (UI/l) | 15 | 35 |
| ALT (UI/l) | 32 | 87 |
| ALP (UI/l) | 19 | 70 |
| GGT (UI/l) | 0.1 | 0.6 |
| Cholinesterase (UI/l) | 1955 | 3950 |
| Total bilirubin (mg/dl) | 0.14 | 0.26 |
| Total Proteins (g/dl) | 6.3 | 7.8 |
| Albumin (g/dl) | 3.0 | 4.0 |
| Globulins (g/dl) | 3.0 | 4.5 |
| Albumin/Globulins ratio | 0.72 | 1.25 |
| Cholesterol (mg/dl) | 95 | 210 |
| Triglycerides (mg/dl) | 19 | 81 |
| Urea (mg/dl) | 32 | 64 |
| Creatinine (mg/dl) | 0.95 | 1.85 |
| Glucose (mg/dl) | 86 | 116 |
| Calcium (mg/dl) | 9.3 | 11.2 |
| Phosphorus (mg/dl) | 3.5 | 6.6 |
| Magnesium (mmol/l) | 0.81 | 1.05 |
| Sodium (mEq/l) | 145 | 152 |
| Potassium (mEq/l) | 3.5 | 4.7 |
| Sodium/potassium ratio | 31 | 43 |
| Chloride (mEq/l) | 112 | 119 |
| Corrected chloride (mEq/l) | 112 | 119 |
| Serum Iron (µg/dl) | 50 | 118 |
| UIBC (µg/dl) | 130 | 225 |
| TIBC (µg/dl) | 175 | 303 |
| Transferrin saturation (%) | 19.5 | 42.5 |
| Serum Amyloid A (µg/ml) | 0.1 | 0.5 |
