# Supplementary material for: Clinical evaluation of outdoor cats exposed to ectoparasites and associated risk for vector-borne infections in southern Italy
Source: Parasit Vectors. 2018 Mar 20;11:136. doi: 10.1186/s13071-018-2725-8 (PMC5859451; doi:10.1186/s13071-018-2725-8)
Supplement: Supplementary file 3 — Table S3. Ordered logistic regression analysis of VBP. (DOCX 14 kb) [file 13071_2018_2725_MOESM3_ESM.docx]

**Additional file 3: Table S3.** Ordered logistic regression analysis of VBP

co-infections

| Variable | Co-infection | |
| --- | --- | --- |
|  | ORs [95% Cl] | P-value |
|  |  |  |
| Adult age | 1.13 [0.54; 2.33] | 0.74 |
| Outdoor life style | 1.64 [0.62; 4.28] | 0.31 |
| Ectoparasiticide treatment | 1.11 [0.42; 2.97] | 0.82 |
| Lymph node enlargement | 1.88 [0.78; 4.50] | 0.15 |
| Skin lesions | 1.56 [0.71; 3.41] | 0.26 |
| FIV antibody positivity | 1.57 [0.37; 6.58] | 0.53 |
| Anemia | 0.67 [0.34; 1.35] | 0.26 |
| Neutrophilia | 1.33 [0.58; 3.012] | 0.49 |
| Monocytosis | 1.00 [0.43; 2.31] | 0.99 |
| High CK | **0.36 [0.19; 0.71]** | **0.00** |
| High ALT | 1.22 [0.30; 4.89] | 0.77 |
| High ALP | 0.61 [0.25; 1.49] | 0.28 |
| Low Albumin | 1.29 [0.62; 2.66] | 0.48 |
| High Globulins | 2.09 [0.95; 4.59] | 0.06 |
| High Creatinine | 0.59 [0.16; 2.18] | 0.435 |
| High Phosphorus | 1.00 [0.46; 2.19] | 0.98 |
| High Serum amyloid A | 1.87 [0.84; 4.13] | 0.12 |

Significant OR (P < 0.05) are shown in bold
